# Endocrine-disrupting chemical exposure during differentiation alters the proliferation–maturation balance in stem-cell islets

**DOI:** 10.1093/toxsci/kfaf163

**Published:** 2025-11-20

**Authors:** June H Gudmestad, Lucas Unger, Joao A Paulo, Luiza Ghila, Thomas A Legøy

**Affiliations:** Department of Clinical Science, Faculty of Medicine, University of Bergen, 5021 Bergen, Norway; Department of Clinical Science, Faculty of Medicine, University of Bergen, 5021 Bergen, Norway; Department of Cell Biology, Harvard Medical School, Boston, MA 02115, United States; Department of Clinical Science, Faculty of Medicine, University of Bergen, 5021 Bergen, Norway; Department of Clinical Science, Faculty of Medicine, University of Bergen, 5021 Bergen, Norway

**Keywords:** endocrine-disrupting chemicals, bisphenols, persistent organic pollutants, induced pluripotent stem cells, stem cell islets, maturation

## Abstract

Exposure to endocrine-disrupting chemicals (EDCs) is increasingly recognized as a risk factor for diabetes, primarily through disruption of pancreatic beta-cell function and insulin signaling. These effects can arise not only from adult exposure but also during development, as many EDCs can cross the placental barrier. However, models that accurately mimic human pancreatic islet development are limited. In this study, we reported the first toxicological application of stem cell islets (SC-islets) to investigate the developmental effect of EDCs. Using human-induced pluripotent stem cells (iPSCs), we generated SC-islets and exposed them to a mixture of bisphenol A, bisphenol S, and trans-nonachlor during differentiation. EDC exposure resulted in SC-islets with an altered transcriptional profile, characterized by reduced expression of beta-cell maturity markers, increased proliferation markers, and elevated KI67-positive cell counts. These features resembled earlier developmental stages and deviated from mature human islet profiles, suggesting a delay in differentiation. Our findings establish SC-islet differentiation as a novel and relevant in vitro model for assessing the developmental toxicity of EDCs, with outcomes consistent with in vivo studies. This model opens new avenues for mechanistic studies and chemical safety assessment in endocrine development.

The global rise of diabetes incidence cannot be fully explained by genetics and lifestyle factors alone. Increasing evidence implicates environmental exposures, particularly to endocrine-disrupting chemicals (EDCs), as significant contributors to metabolic disorders, including diabetes (Duh-Leong et al. 2023). EDCs such as bisphenol, phthalates, and persistent organic pollutants have been shown to interfere with glucose homeostasis, insulin signaling, and pancreatic beta-cell function (La Merrill et al. 2020). Epidemiological studies have linked higher body burdens of these chemicals with increased risk of Type 2 diabetes (T2D) (Gore et al. 2015). For example, there are legacy compounds like *trans-nonachlor* with a background exposure that is ever-present in the human population. This compound is linked to the development of T2D (Lin and Yin 2023) and can be passed from mother to fetus (Neta et al. 2011). Bisphenol A (BPA) and bisphenol S (BPS) are plasticizers to which most of the population is exposed via digestion and dermal contact. They have the ability to cross the blood–placenta barrier and are linked to the development of T2D and beta-cell failure (Liu et al. 2017; Alharbi et al. 2022; Daian et al. 2023; Estévez-Danta et al. 2024). There are multiple potential mechanisms that can lead to beta-cell failure giving rise to diabetes, like ER stress, oxidative stress, mitochondrial dysfunction, dedifferentiation, or transdifferentiation. These cause beta-cell mass reduction and beta-cell dysfunction (Son and Accili 2023). To map these decay mechanisms, we are dependent on good models that can mimic exposure of the human endocrine islets.

Multiple immortalized beta-like cell lines of murine origin have been created, which are useful for large-scale toxicant screenings due to their ability to proliferate while also presenting some glucose responsiveness (Poitout et al. 1996). This apparent advantage is likely also their weakness, as beta-cells in vivo have a low ability to proliferate (Gregg et al. 2012). Some of these in vitro models have also been challenged as a diabetogenic pollutant screening system, e.g. the INS-1 832/12 cell line that did not respond as anticipated to chronic experiments based on in vivo findings (Hectors et al. 2013). It is possible to use primary rodent islets to overcome some of the drawbacks of immortalized rodent beta-cell lines. However, there are some key differences between rodent and human islets that could impact translatability, like mice and rats expressing 2 insulin genes, whereas humans express one (Irwin 2021), and the significant differences in islet development, architecture, and composition (Dolenšek et al. 2015). A better model would be bona fide human islets; however, access to them is scarce, and culturing negatively affects their viability and functionality (Hart and Powers 2019).

An in vitro model that was meant to bridge the gap between rodent models and the lack of human islets is the EndoC-βH1 cell line (Scharfmann et al. 2014), a human insulin cell line that has low proliferation capabilities but is glucose responsive (Tsonkova et al. 2018). Yet, the limited proliferative capacity poses a significant challenge for large-scale screening efforts. Another model that is helping to bridge this gap, while holding the potential for up-scaling, is the use of embryonic or induced pluripotent stem cell (ESCs or iPSCs)-derived pancreatic endocrine-like cells. These cells are widely used in diabetes research and are increasingly being explored as a potential source for beta-cell replacement therapy, offering an alternative to human islets (Hogrebe et al. 2023). By treating the stem cells with a cocktail of chemicals at different stages, the cells are guided stepwise through development toward islet endocrine cells (Balboa et al. 2022), offering a robust and scalable source of pancreatic endocrine-like cells.

Interestingly, even though ESC- and iPSC-derived pancreatic endocrine cells are widely used in the field of diabetes, their use for toxicological screening remains limited. Notably, Zhou et al. (2018) demonstrated that the pesticide propargite induces beta-cell death in a gene-dependent manner, using a human stem cell-based model (Zhou et al. 2018). However, this remains one of the few examples of such a direct exposure study. Follow-up studies have largely focused on neurotoxicity or disease modeling, with limited expansion on the beta-cell toxicology platform. This underscores a critical gap in the systematic evaluation of environmental toxicants using stem cell-derived islet models. In addition, this study focused on exposure to a single compound, whereas a real-world exposure often involves low doses of multiple compounds that may interact synergistically, additively, or antagonistically, effects that are frequently missed in single-compound studies (Martin et al. 2021). Further on, as the differentiation protocol offers a stepwise differentiation, it also opens up for assessing potential development effects of chemical exposure, something that has been called for (MacFarlane and Bruin 2021).

To address this research need, we employed a well-established differentiation protocol to generate mature stem cell islets (SC-islets) (Balboa et al. 2022) and assess the impact of a mix of EDCs during differentiation to elucidate the developmental exposures’ impact on the maturation of SC-islets.

## Materials and methods

### Human-induced pluripotent stem cell culturing

The human-induced pluripotent stem cell (hiPSC) used in this study was commercially bought from Synthego, donor PGP1. They were created by retroviral reprogramming of donor skin fibroblasts from the Personal Genome Project (Coriell, GM23338). The hiPSCs were cultured on Geltrex LDEV-free reduced Growth Factor (Gibco, A1413202)-coated plates and maintained in mTeSR Plus cGMP stabilized feeder-free maintenance medium (Stem Cell Technologies, 100-0276). For passaging, the colonies were treated with Gentle Cell Dissociation Reagent (Stem Cell Technologies, 100-0485) and mild fragmentation of colonies. All cultures tested negative for mycoplasma prior to differentiation by using MycoAlert Mycoplasm Detection Kit (Lonza, LT07-418).

### In vitro differentiation and EDC treatment

The hiPSCs were differentiated toward SC-islets following previously published stepwise protocol (Balboa et al. 2022; Barsby et al. 2022; Unger et al. 2024); see Tables S1 to S3 for detailed differentiation cocktails. For differentiation, 12 million cells were seeded in a 10-cm dish. When reaching S4d3, each 10-cm dish was transferred into 2 wells of a 6-well Aggrewell plate (Stem Cell Technologies, 34425), with 6 million cells in each well. These 2 wells were divided into the control (NT) and EDC-treated groups to minimize differentiation differences. EDC treatment was initiated when the aggregates were transferred from Aggrewell to ultra-low attachment (ULA) plates at Stage 5, Day 3. The EDCs were diluted in DMSO and added in the media to a final DMSO concentration of 0.1% and a concentration of 1 nM of BPA, BPS, and trans-nonachlor. This dose is a human-relevant dose, where the trans-nonachlor concentration is at the higher end of the range detected in human whole blood (Mangum et al. 2016), and the BPA and BPS are within the lower range of what is detected in whole blood (Li et al. 2022) and cord blood (Gély et al. 2021). The treatment was performed for 14 consecutive days, with media change every second day. The NT wells were treated with 0.1% DMSO. After 14 days, the SC-islets were maintained in standard S7 media until collection at Day 38, or S7 at Day 14.

### Immunofluorescence

SC-islets from 4 separate rounds of differentiation for each condition were washed with DPBS and fixed in 4% Paraformaldehyde (PFA) overnight at 4 °C. After fixation, SC-islets were washed 5 times with DBPS and dehydrated using 20% and 30% sucrose gradients. The SC-islets stayed in 30% sucrose overnight at 4 °C. The next day, the fixed, dehydrated SC-islets were preincubated in Tissue-Tek O.C.T. compound (Sakura Finetek, 4583) for 1 h before freezing in fresh OCT. The OCT blocks were sectioned into 5 μm sections using a cryotome (Leica) and adhered to gelatine-coated microscope slides. Prior to antibody staining, sections were rehydrated with PBS, treated with 2% Triton X-100 (Sigma Aldrich, X-100) for permeabilization, and unspecific binding was blocked by washing with 2% BSA for 1 h at room temperature. Primary antibody staining was performed by overnight incubation at 4 °C with the primary antibody mix diluted in 2% BSA. The primary antibodies used were Rat anti-C-peptide (DSHB, GN-ID4, 1:250), Guinea pig anti-glucagon (Geneve Antibody Facility, ABCD_AK247, 1:400), and rabbit anti-Ki67 (Abcam, ab15580, 1:250). After 3 short washes with PBS, the sections were stained with secondary antibodies for 1 h at room temperature. The secondary antibodies used were Donkey anti-rat Alexa 488, Goat-anti guinea pig Alexa 546, and Donkey anti rabbit Alexa 647 (Invitrogen). After 3 new short washes with PBS, and 1 wash with ddH_2_O, the slides were quickly dried and mounted using Aqueous Mounting Medium with DAPI (Abcam, AB104139). The sections were imaged using a fluorescence microscope, Leica TCS SP8 STED 3X (Leica Microsystems, Wetzlar, Germany), using a 40× objective. The number of hormone-positive cells was counted using an automated counting software we have previously published (Unger et al. 2025), whereas nuclear KI67 staining was counted manually.

### Glucose-stimulated insulin secretion

Glucose-stimulated insulin secretion (GSIS) was performed on SC-islets from 6 separate differentiations for each condition. Twenty-four hours prior to the GSIS test, approximately one-third of the SC-islets were transferred to new ULA 6-well plates containing treatment media devoid of ITS-x to eliminate exogenous insulin. All incubation steps were conducted in a humidified incubator at 37 °C with 5% CO_2_. After 24 h, 1 ml of media was collected, and the SC-islets were transferred to a 12-well plate pre-treated with anti-adhesion solution (StemCell Technologies) and washed twice with KREBS buffer (129 mM NaCl, 5 mM NaHCO_3_, 2.5 mM CaCl_2_, 1.2 mM MgSO_4_, 1 mM Na_2_HPO_4_, 1.2 mM KH_2_PO_4_, 10 mM HEPES, 0.3% BSA, pH 7.4). The SC-islets were primed for 30 min with KREBS buffer containing 1.67 mM glucose, followed by incubation with 1.67 mM glucose KREBS buffer for 90 min. Media was collected, and the SC-islets were incubated with 1 ml of 20 mM glucose KREBS buffer for 90 min. Media was collected, and the SC-islets were washed once with 1 ml of 1.67 mM glucose, followed by incubation with 1 ml of 1.67 mM glucose for 60 min. The media was collected, and finally, the SC-islets were incubated with 1 ml of 30 mM KCl KREBS buffer for 60 min. This media was collected, and the SC-islets were lysed by adding 75% ethanol with 1.5% HCl and sonication at 30% power for two 20-s intervals. DNA concentration was measured using a Nanodrop One spectrophotometer (ThermoFisher Scientific) and used for normalization. Insulin secretion was measured by Insulin ELISA (Mercodia) of the media, following the manufacture’s protocol, and the absorbance was measured using Infinity 200 Pro (Tecan).

### RNA sequencing

#### RNA extraction

SC-islets from 3 separate differentiation rounds for each condition were washed with DPBS and frozen in RLT buffer with 1% 2-mercaptoethanol. RNA was extracted using the RNeasy Mini Kit (Qiagen, Redwood City, CA, USA; 74104) from lysed cells by following the protocol from the manufacturer. The RNA was eluted with 30 μl of nuclease-free water, and quality control and concentration were measured using RNA ScreenTape (Agilent, 5067-5576) on TapeStation 4150 (Agilent G2992AA).

#### Sequencing

The sequencing was performed by the UiB Genomics Core Facility. Library preparation was performed by using the Illumina Stranded mRNA Ligation Kit, followed by quantification using KAPA qPCR quantification. Library quality control was performed using Agilent TapeStation. Standard workflow methods were used to load the NovaSeq SP flowcell and sequenced 2×100 bp paired-end reads for Illumina NovaSeq 6000, 40 million reads per sample.

#### Data and pathway analysis

The FastQ files from sequencing were processed in the CLC Genomics Workbench 24.0 (Qiagen, Aarhus, Denmark). Pre-processing included adapter and quality score-based trimming, using the default setting provided by the trimming tool in the CLC software. Alignment, quantification, and differential gene expression analysis were carried out using the RNA-Seq and Differential Gene Expression Analysis (v1.2) provided by the CLC Workbench. For alignment, we used the reference genome GRCh.38.p14. The differentially expressed gene (DEG) lists were uploaded to Ingenuity Pathway Analysis (Qiagen, version 127006219) for further analysis (Krämer et al. 2014). Only DEGs with a 2-log fold change lower or higher than 0.3 and *P*-value lower than 0.5 were included for pathway prediction, and network settings were kept at standard.

### Proteomics

#### Protein extraction and peptide preparation

Three separate SC-islets from EDC treatment and 4 NT differentiation rounds were washed with DPBS, harvested, centrifuged at 300×*g* for 4 min, and resuspended in 80 μl 8M Urea 200 mM EPPS pH 8.5 protease inhibitor with EDTA. The samples were sonicated 3 times for 30 s to lyse the cells. The samples were centrifuged at 14,000×*g* for 3 min, and the supernatant was collected. Protein concentration was determined using the BCA assay, and 100 μg of protein from each sample was processed; 25 mM Tris and 0.5 mM CaCl_2_ were added to each sample and slowly mixed for 5 min; 9 mM dithiothreitol was added to each sample and incubated for 1 h. Afterwards, 20 mM iodoacetamide was added and incubated for 1 h in the dark. To quench the reaction, 1.6 mM dithiothreitol was added and incubated for 10 min. Before trypsinization, 35 mM Tris 0.7 mM CaCl_2_ was added, and 2 μg of trypsin and left at 37 °C overnight on a shaker. To stop the reaction, 0.8% formic acid (FA) was added. All peptides were desalted by Oasis plate, C18 column (Waters).

#### TMT labeling and fractionation

The TMT labeling and fractionation protocol was performed as previously described (Legøy et al. 2020a; Liu et al. 2023); 25 μg of peptides from each sample was used for subsequent processing and dissolved in 40 μl of TEAB; 500 μg of TMTpro 16 plex reagents were dissolved in 75 μl acetonitrile; and 12 μl of TMTpro 16 plex reagent was added to the samples of one 16 plex and incubated for 1 h. The reaction was quenched by adding hydroxylamine to a final concentration of 0.3%. All the TMT-labeled samples across 1 plex were pooled before desalting using an Oasis plate. The samples were frozen at −80 °C before vacuum centrifugation to near dryness. The TMT-labeled samples were first fractioned using Thermo Scientific Pierce Strong Anion Exchange Spin Columns (Fisher Scientific, 10311514) by using 20 mM NH_4_OH and 15% acetonitrile in combination with 0, 20, 50, 100, 250, or 500 mM ammonium acetate, creating 6 fractions. Fractions 0, 20, and 500 ammonium acetate were pooled, as well as fractions 100, 250, and 500 mM were a second pool. Each pool was frozen at −80 before being vacuum-centrifuged to near dryness. Each pool was desalted using StageTip (Fisher Scientific, 87784). Each fraction pool was subsequently fractionated using the Thermo Scientific Pierce High pH Reversed-Phase Peptide Fractionation Kit (Fisher Scientific, 15362617). The fractions were collected by adding 0.1% triethylamine with 7.5%, 10%, 12.5%, 15%, 17.5%, 20%, 22.5%, 27.5%, 30%, 35%, and 60% acetonitrile. The following fractions were pooled: 7.5 and 22.5; 10 and 25; 12.5 and 27.5; 15 and 30; 17.5 and 35; 20% and 60% acetonitrile; and 0.1% triethylamine. Giving a total of 6 fractions per sax fraction, leading to a total of 12 fractions per 16-plex. Each fraction was frozen and vacuum-dried to near dryness before desalting with StageTip.

#### LC-MS/MS and proteomics data analysis

The mass spectrometry and subsequent data analysis were performed as previously described (Mathisen et al. 2023). In short, approximately 5 μg from each of 12 non-adjacent fractions was dissolved in 5% aqueous FA/5% acetonitrile prior to LC-MS/MS analysis on an Orbitrap Eclipse mass spectrometer (Thermo Fisher Scientific, San Jose, CA) coupled to a Neo Vanquish liquid chromatography (LC) pump (Thermo Fisher Scientific), and a scan sequence using MS1 spectrum was performed. Data were acquired using the FAIMSpro interface. Mass spectra were processed using a Comet-based in-house software pipeline, and spectra were converted to mzXML using a modified version of ReAdW.exe. Database searching included all entries from the human Uniprot database (March 2021). Protein assembly was guided by principles of parsimony to produce the smallest set of proteins necessary to account for all observed peptides. Proteins were quantified by summing reporter ion counts across all matching peptide spectrum matches using in-house software. Each reporter ion channel was summed across all quantified proteins and normalized, assuming equal protein loading of all samples. For proteomics data, we used Metabolomic Data Analysis with MetaboAnalyst 6.0 (Pang et al. 2024) for statistical analysis. Data were first normalized to the sample median before log10 data transformation and autoscaling of the data. For differentially abundant proteins (DAPs), a log2 fold change of 0.3 was set and a *P*-value < 0.05 following a *t*-test.

### Statistical analysis

Statistical analyses were performed using GraphPad Prism v10.5.0 (GraphPad Software Inc., USA). For immunofluorescence data, we used the parametric Welch’s *t*-test and displayed the data as average ± SD. Statistical significance was defined as *P* < 0.05.

## Results

### Efficient generation of SC-islets including all the main islet endocrine populations

**Fig. 1.**
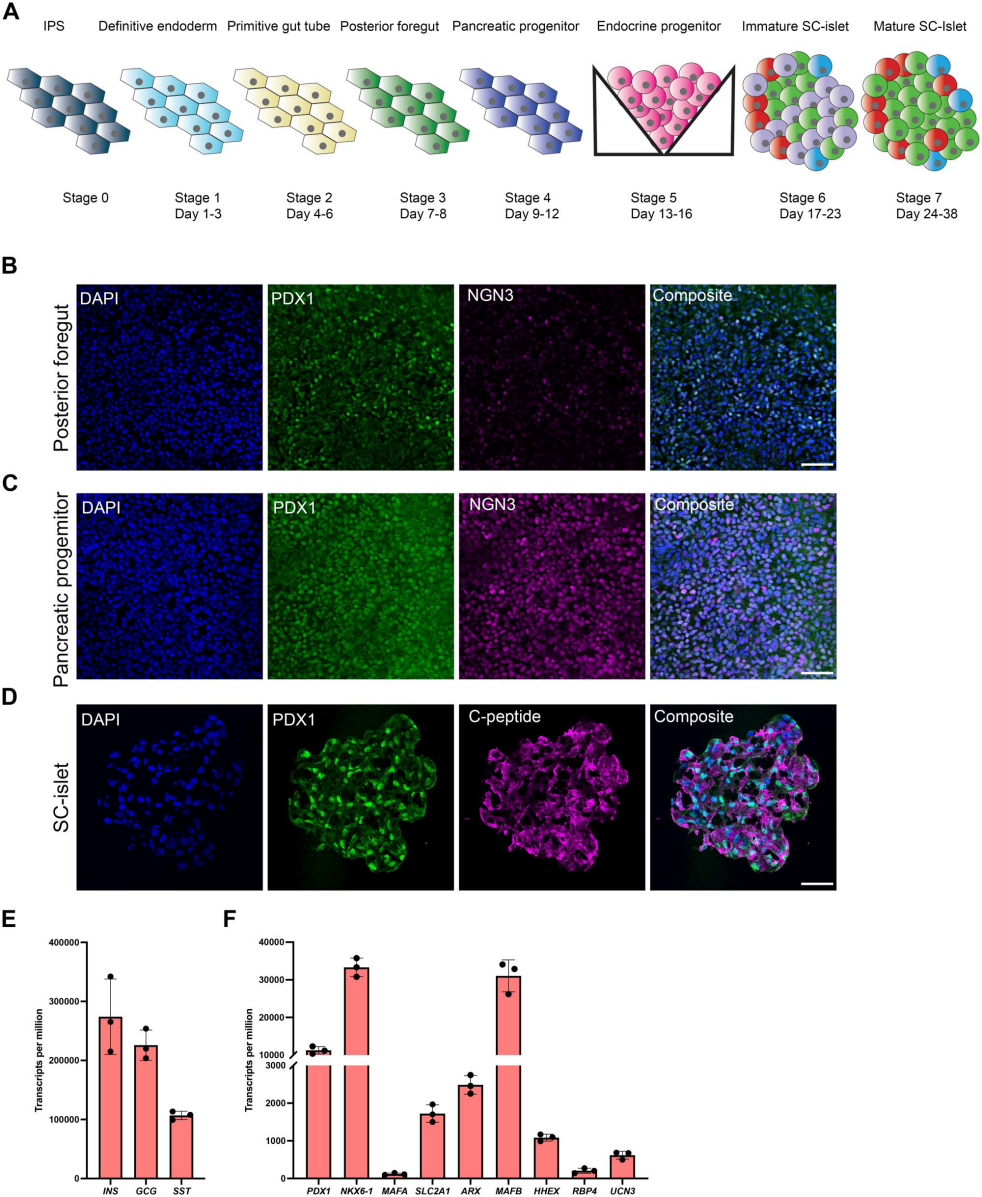
Differentiation toward SC-islets. A) Schematic representation of the stepwise differentiation protocol from iPSCs to SC-islets. B to D) Representative immunofluorescence images of differentiation stages, DAPI (blue), PDX1 (green), and NGN3 (magenta) staining for B) posterior foregut stage and C) pancreatic progenitor stage. D) SC-islets at S7d14 stained for DAPI (blue), PDX1 (green), and C-peptide (magenta). Scalebar 50 µm. Average transcripts per million (RNAseq) from S7d14 SC-islets of E) endocrine hormones and F) endocrine cell maturity markers. Graph data are shown as average ± SD.

By using a 7-stage stepwise protocol developed by Barsby et al. (2022), we generated functional pancreatic SC-islets from hiPSCs with a metabolic profile mimicking human islets (Fig. 1A). During the differentiation, PDX1- and NGN3-positive progenitor cells arose at the posterior foregut (Fig. 1B) and pancreatic progenitor (Fig. 1C) stage. Both NGN3 and PDX1 are expressed in the developing pancreas and are necessary to form pancreatic endocrine cells, where PDX1 expression is maintained in beta cells, whereas NGN3 expression is lost in adult cells (Gradwohl et al. 2000; Mellitzer et al. 2006; Gao et al. 2014; Ebrahim et al. 2022). At the endocrine progenitor stage, aggregates were formed, which maintained the PDX1 expression in addition to initiating production of pancreatic hormones, such as insulin, at the SC-islets stage (Fig. 1D). The resulting SC-islets were composed of several cell types expressing the main islet hormones insulin, glucagon, and somatostatin (Fig. 1E). In addition, these cells expressed key maturity markers for each of the main islet endocrine populations, such as *PDX1*, *NKX6-1*, *SLC2A1*, *MAFA*, and *MAFB* (insulin-producing beta cells); *ARX* (glucagon-producing alpha-cells); *HHEX* and *RBP4* (somatostatin-producing delta-cells); and the pan-endocrine marker *Urocortin 3* (*UCN3*) (Fig. 1F).

### EDC exposure affects maturity markers but not hormonal cell fate acquisition

**Fig. 2.**
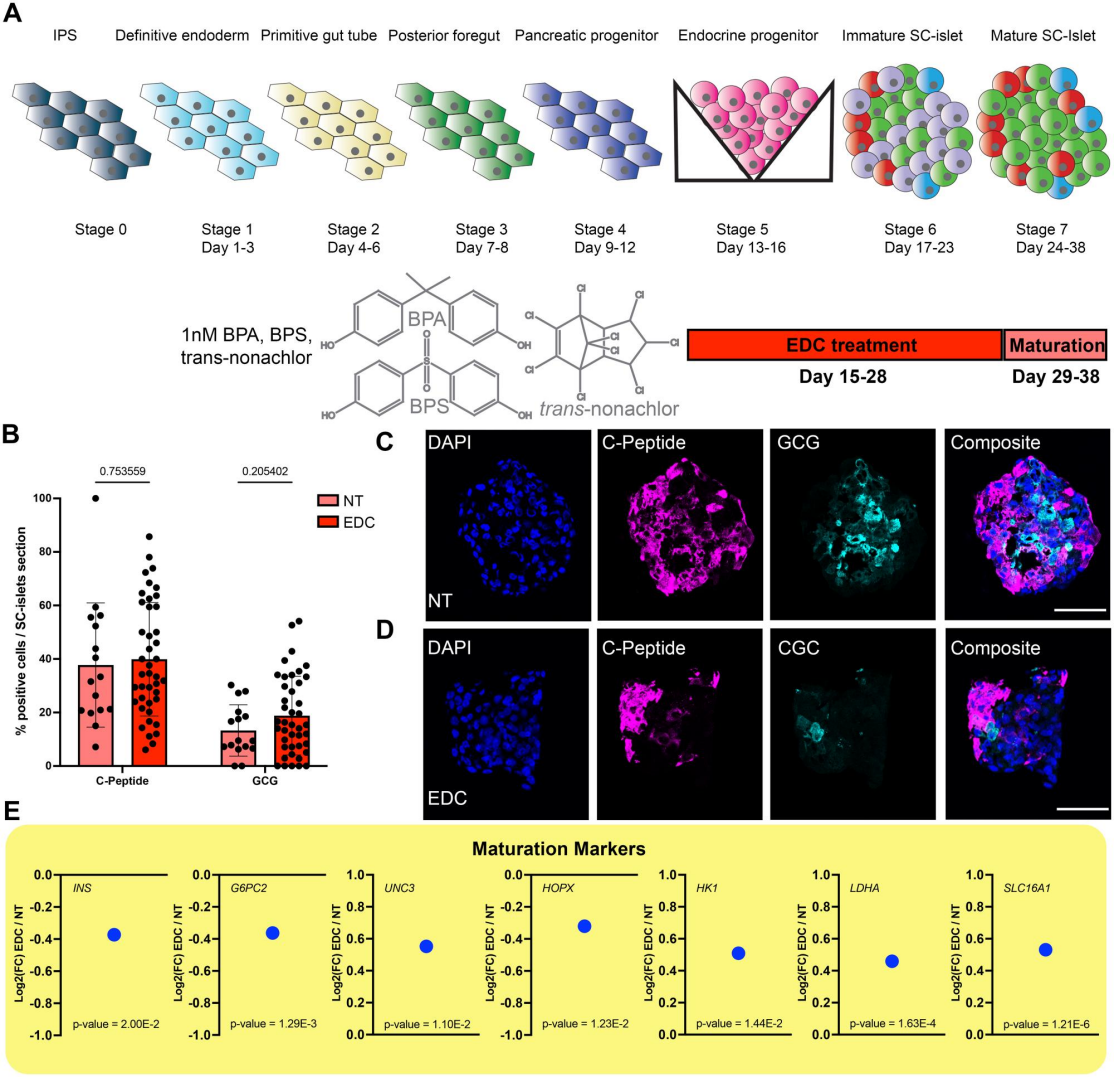
EDC treatment affect maturation but not hormone fate acquisition. A) Schematic representation of the stepwise differentiation protocol including timing of EDC treatment. B) Percentage of cells per SC-islets section expression insulin or glucagon in NT- and EDC-treated group, graphs show average values ± SD, dots represent individual SC-islets sections, *n* = 4. C and D) representative immunofluorescent staining of a SC-islet section stained with DAPI (blue), C-peptide (magenta), and glucagon (cyan) for NT- and EDC-treated group. Scalebar 50 µm. E) Graphs showing DEGs (RNAseq) of maturation markers represented as log2 foldchange between EDC and NT group.

To assess the developmental impact of exposure to the EDCs BPA, BPS, and trans-nonachlor during endocrine progenitor and initial SC-islet maturation, we initiated the EDC treatment at Day 15 of the differentiation protocol. This timepoint represents the endocrine progenitor stage and the timepoint in the protocol when the aggregates are formed. We chose this timepoint to avoid altering the aggregate formation, as this is a key step in the protocol. After 14 days of treatment, the SC-islets were maintained without treatment for an additional 10 days before collection (Fig. 2A). These last days of maturation are key in reaching functional SC-islets (Balboa et al. 2022). We observed that the EDC treatment did not affect the hormonal cell fate acquisition by performing immunofluorescence analysis of SC-islet sections between the different treatments. There was no significant difference observed between the percentage of insulin cells (EDC vs NT values) nor glucagon cells (EDC vs NT values) (Fig. 2B to D). However, hormone production alone is considered insufficient for assessing functional maturation. Therefore, we performed RNA sequencing (RNAseq) to assess the level of alterations in the gene expression landscape after EDC exposure. We observed a downregulation of key genes for the function of beta cells (Fig. 2E), like *insulin* (*INS*) and *glucose-6-phosohatase catalytic subunit 2* (*G6PC2*), or maturation like *HOP Homeobox* (*HOPX*) (Hrvatin et al. 2014; Balboa et al. 2022). Interestingly, we found an upregulation of *UCN3*, which is produced by both alpha- and beta cells to promote SST release (van der Meulen et al. 2015). In addition, we found a set of genes that are considered disallowed in human islets, such as hexokinase I (*HK1*), *lactate dehydrogenase isoform A* (*LDHA*), and monocarboxylate transporter 1 (*SLC16A1*) (Balboa et al. 2022), to be upregulated. These findings indicated that even though SC-islets with similar hormone composition were formed, exposure to EDC during differentiation impairs the transcriptional landscape connected with maturation and probably functionality.

### EDC exposure promotes cell cycle and proliferation signaling

**Fig. 3.**
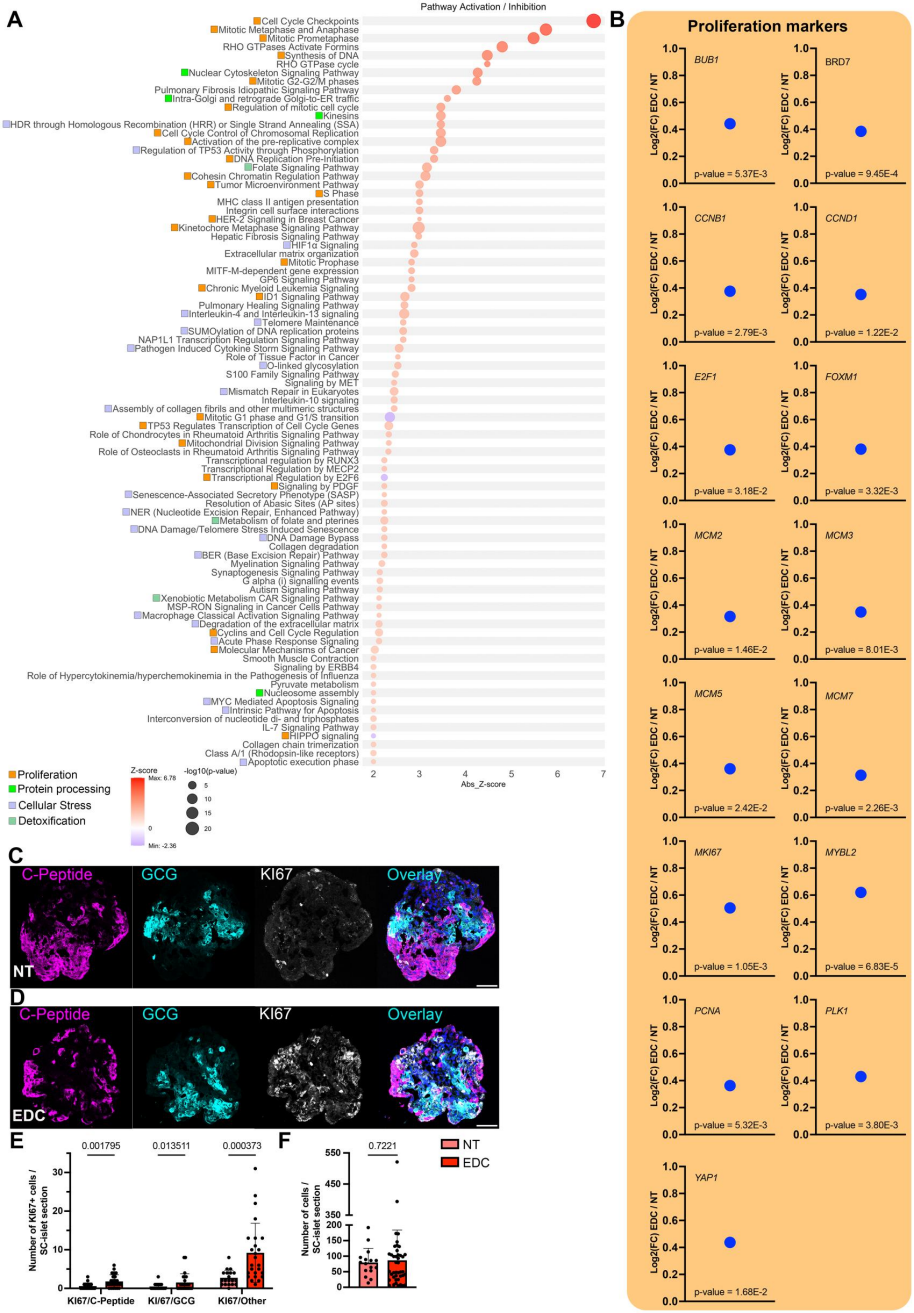
Proliferation signals are increased after EDC exposure. A) All significant upstream regulators of DEGs between EDC and NT, −log(*P*-value) > 1.3 and absolute *z*-score ≥ 2. Pathways are categorized into proliferation, protein processing, cellular stress, and detoxification by colored boxes. B) Selected genes connected with proliferation signatures from RNAseq, differentially expressed between EDC and NT. C and D) Representative immunofluorescence images of SC-islets stained for C-peptide (magenta), glucagon (cyan), KI67 (gray), and DAPI (blue), scale bar 50 µm, for C) NT and D) EDC treated. E) Graph showing number of C-peptide, glucagon, or other cells per SC-islets co-expressing KI67 in NT and EDC, *n* = 4. F) Graphs showing total number of cells per SC-islet section in NT and EDC, *n* = 4.

By analyzing the transcriptional landscape by pathway analysis of DEGs between the EDC and NT groups, we observed that some of the main pathways affected by the EDC treatment were related to cell cycle and proliferation. All major steps of cell cycle progression, including the G1/S phase transition and other cell cycle-related functions, showed increased predicted activation scores with a z-score greater than 2 (Fig. S1A). Additionally, several functions associated with proliferation, such as tumor proliferation and colony formation, were predicted to be activated. Notably, the function “Cell proliferation of tumor cell lines” was supported by 270 molecules identified in our DEGs list (Fig. S1B), accompanied by the enrichment of canonical pathways involved in cell cycle progression and proliferation (Fig. 3A, orange boxes). Multiple key proliferation markers were found upregulated, like multiple minichromosomal maintenance complex components; *Mitotic checkpoint serine/threonine-protein kinase BUB1* (*BUB1*, Log2(FC) = 0.442); *marker for proliferation Kiel 67* (*MKI67*, Log2(FC) = 0.552); proliferating cell nuclear antigen (*PCNA*, Log2(FC) = 0.363); and *yes-associated protein 1* (*YAP1*, Log2(FC) = 0.437) (Fig. 3B). Pathway analysis revealed regulatory networks highlighting MYC as a key upstream regulator of proliferation markers (Fig. S1C). The transcriptomic proliferative landscape was translated into the protein level in the EDC-treated SC-islets, with an increased number of EDC-treated SC-islets cells expressing MKI67 (Fig. 3C and D). There was a slight increase in the number of C-peptide- and GCG-expressing cells co-expressing MKI67, whereas we observed an elevated increase in the non-insulin and non-glucagon (insulin and glucagon negative) cells expressing MKI67 (Fig. 3E). Notably, at this timepoint, the increase in proliferation did not cause any changes in the total number of cells counted per SC-islet section (Fig. 3F). EDC treatment during differentiation clearly upregulates a transcriptional landscape connected with proliferation, which is translated into an increased number of cells expressing MKI67 at the protein level.

### Proliferation signals are accompanied by protein processing

**Fig. 4.**
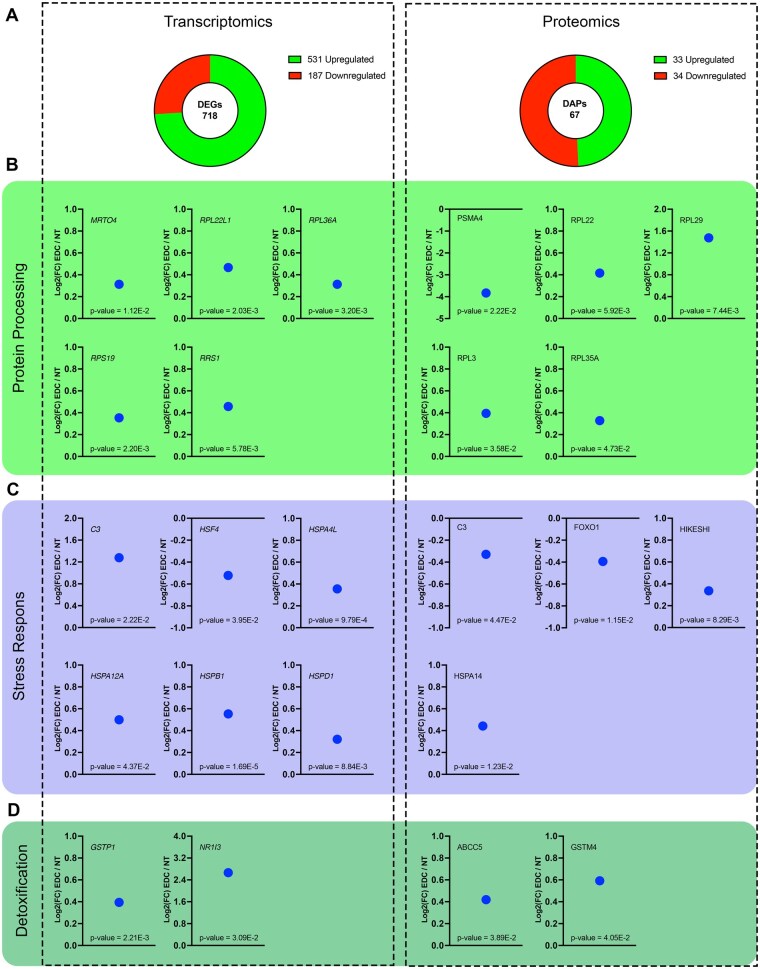
Integrated transcriptomic and proteomic analysis of protein modification, cellular stress response, and detoxification pathway. A) Total number of up- and downregulated DEGs (left) and DAPs (right) between EDC and NT groups. B) Graphs showing selected genes and proteins connected with protein processing. C) Selected genes and proteins connected with cellular stress response showed in graphs. D) Graphs showing selected genes and proteins connected with detoxification. All graphs show log2 fold change between EDC and NT based on RNAseq (left) and proteomics (right).

We further assessed the protein landscape, as proliferation is usually associated with increased protein production (Zhu and Thompson 2019). We compared our transcriptomics with proteomics of EDC vs NT. We found a total of 718 DEGs (531 upregulated and 187 downregulated) and 67 DAPs (33 upregulated and 34 downregulated) (Fig. 4A). For protein processing, we found upregulation of multiple genes and proteins for the ribosome, like upregulation of *ribosomal protein L22 like 1* (*RPL22L1*, Log2(FC) = 0.466), *ribosomal protein L36 A* (*RPL36A*, Log2(FC) = 0.313), ribosomal protein S19 (*RPS19*, Log2(FC) = 0.353) as transcript, and ribosomal protein L22, L29, and L35A (*RPL22*, Log2(FC) = 0.415, *RPL29*, Log2(FC) = 1.475, *RPL35A*, Log2(FC) = 0.328) on protein level. In addition, we found upregulation of ribosomal assembly marker MRT4 Homolog, Ribosome Maturation Factor (*MRTO4*, Log2(FC) = 0.313) transcripts and downregulation of protein turnover protein Proteasome 20S Subunit Alpha 4 (*PSMA4*, Log2(FC) = −3.831) (Fig. 4B). Pathway analysis of the DEGs in the EDC-treated group found canonical pathways linked to cytoskeletal signaling and protein processing to be activated (Fig. 3A, light green boxes). These findings combined indicate an increased protein machinery and cytoskeletal signaling and organization to support the transcriptional proliferative landscape.

### EDC exposure causes prolonged stress and detoxification response

Even though the SC-islets were not exposed to EDCs for the last 10 days of differentiation and maturation, they showed an increase in certain stress and detoxification responses both at the transcript and protein levels (Fig. 4B and C). One group of proteins known to be activated by cellular stress is the heat shock proteins. They become activated as a response to cellular stress to protect against misfolding of proteins (Wan et al. 2020). We observed a significant increase in the abundance of heat shock proteins like Heat Shock Protein A14 (HSPA14, Log2(FC) = 0.442) at the protein level and the upregulation of multiple transcripts like *Heat Shock Protein Family A (Hsp70) Member 4 Like* (*HSPA4L*, Log2(FC) = 0.355), *Heat Shock Protein Family A (Hsp70) Member 12A* (*HSPA12A*, Log2(FC) = 0.499), *Heat Shock Protein Family B (Small) Member 1* (*HSPB1*, Log2(FC) = 0.553), and *Heat Shock Protein Family D (Hsp60) Member 1* (*HSPD1*, Log2(FC) = 0.321). In addition, we found an upregulation of the heat shock protein translocator Heat Shock Protein Nuclear Import Factor Hikeshi (HIKESHI, Log2(FC) = 0.336), which facilitates the translocation of HSP70 to the nucleus (Kose et al. 2012). In contrast, we observed a downregulation of the Heat Shock Factor 4 (HSF4, Log2(FC) = −0.522), which is one of the regulators of heat shock proteins, yet not in response to cellular stress (Åkerfelt et al. 2010). The SC-islets treated with EDCs had an activated stress response; in addition, they might be less able to handle cellular stress due to a potential reduced stress response pathway by reduced expression of Forkhead Box O1 (FOXO1, Log2(FC) = −0.394). The canonical pathway analysis from IPA, analyzed on the RNAseq data, confirmed signs of cellular stress, with pathways connected with protection against DNA and protein damage being upregulated, in combination with apoptotic pathways, among others (Fig. 3A, lavender blue box). The observed cellular stress systems were accompanied by an activation of detoxification systems, likely as a response to the chemical stress from EDC exposure (Fig. 4D). The SC-islets activated phase II detoxification enzymes like Glutathione S-transferase Pi 1 (GSTP1, Log2(FC) = 0.394) and Glutathione S-transferase Mu 4 (GSTM4, Log2(FC) = 0.592), and the nuclear receptor CAR (NR1I3, Log2(FC) = 2.662) and the transporter ATP-Binding Cassette Subfamily C Member 5 (ABCC5, Log2(FC) = 0.419). The pathway analysis picked up the CAR signaling pathways as being upregulated, as well as folate metabolism and signaling (Fig. 3A, dark green boxes), where folate is necessary for working glutathione detoxification mechanisms (Reed et al. 2008). A TPM value of more than 1 is considered meaningful gene expression of a gene within a sample (Li and Dewey 2011). With this in mind, most of the traditional detoxification machinery is not transcribed in the EDC-treated SC-islets (Fig. S2), and it also removed NR1I3 as a candidate for the detoxification system, which is not surprising given that it is mainly expressed in the liver (Bae et al. 2021). These findings show that even with a 1 week recovery period, the SC-islets have an activated stress and detoxification response to the EDC treatment.

### EDC exposure during differentiation delays maturation

**Fig. 5.**
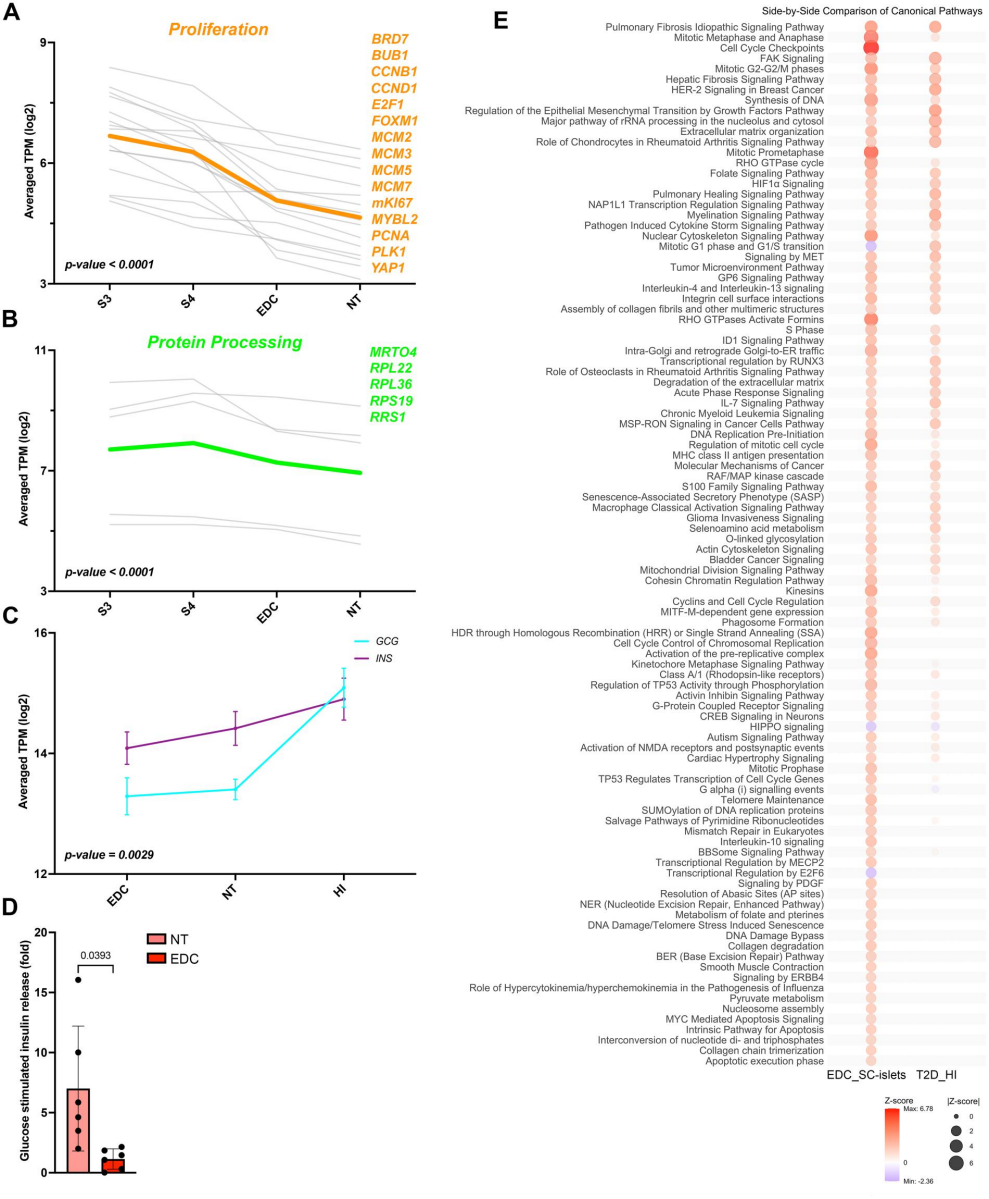
EDC exposure during differentiation delays maturation. A) Graphs showing the averaged (orange) log2 TPM values of selected proliferation markers from Stages 3 and 4 and SC-islets from NT and EDC islets, or B) for selected protein processing markers (green). C) Average log2 TPM ± SD for insulin and glucagon values in NT, EDC, and human islets. The statistical test is Tukey’s two-way ANOVA. D) Graphs showing glucose-stimulated insulin response of SC-islets from NT or EDC as a fold difference between stimulated (20 mM glucose) and basal (1.67 mM glucose) stimulation, statistical test, and Welch *t*-test. E) Comparison of canonical pathways between EDC-treated SC-islets and human islets from Type 2 diabetic patient, with comparison performed using Analysis Match in IPA, comparing all canonical pathways with absolute z-score ≥2 in the SC-islet_EDC data.

To assess how the EDC exposure during differentiation affected the transcriptional landscapes’ push toward SC-islets, we compared the NT and EDC with less mature stages of S3 (posterior foregut) and S4 (pancreatic progenitor) of the differentiation, as well as with transcripts of human islets from donors. Interestingly, we observed that the proliferation (Fig. 5A) and protein processing (Fig. 5B) of the EDC-treated samples were closer to earlier stages of differentiation, whereas the hormone transcriptional landscape of *INS* and *GCG* was further from human islets than the NT counterpart (Fig. 5C). In line with these findings, following a study by Hrvatin et al. (2014), we observed an activation of upstream transcription regulators connected with fetal pancreas, like Fos Proto-Oncogene (FOS), SRY-Box Transcription Factor 4 (SOX4), and SRY-Box Transcription Factor 4 (SOX11) (Fig. S1D), and an inactivation of upstream regulators connected with adult insulin cells like SIX Homeobox 2 (SIX2) and Tumor Protein P53 (TP53) (Fig. S1E). This delay in maturation led to a blunted glucose-stimulated insulin response (Fig. 5D), a key sign of immaturity in SC-islets, especially the insulin-producing cell compartment. Interestingly, by using the Analysis Match function in IPA, we found an overall z-score of 23.87 when matching with a dataset comparing human islets from Type 2 diabetic patients with healthy controls (Project GSE41762, published in Mahdi et al. [2012]), with comparable upstream regulator activation as in our dataset (Fig. 5E). These data combined show that the observed increase in proliferation and protein modification profile relates to reduced maturation and function of SC-islets caused by EDC exposure.

## Discussion

In this study, we demonstrated for the first time that exposure to EDCs during the differentiation of SC-islet leads to increased delayed maturation of insulin-producing cells as well as increased proliferation. These findings reveal that differentiation of iPSCs-derived SC-islets can be used to assess vulnerability of the developing endocrine pancreas to environmental toxicants. By modeling EDC exposure during in vitro differentiation, we provided a human-relevant platform to investigate how early life chemical insults may impair beta-cell development and contribute to long-term metabolic dysfunction.

Even though differentiation of hiPSCs toward pancreatic endocrine islets has the potential to be a great model to study the impact of chemical exposure on islet development and function, it is not utilized. The main focus of the study by Zhou et al. (2018) was the insulin-producing beta cells, and the ESCs or iPSCs differentiation was performed entirely in planar culture conditions (Zhou et al. 2018). It is now well-established that adding a 3D cluster step in the differentiation protocol is key to generate glucose-responsive SC-islets that closely mimic human islets (Balboa et al. 2022). The 3D environment not only creates a more natural spherical environment but it also creates a more islet-like environment with clusters containing the beta cells together with glucagon-producing alpha cells and somatostatin-producing delta cells. Additionally, it is well established that proper beta-cell function is dependent on intra-islet signals between the endocrine cells in the pancreas (Lammert and Thorn 2020). This multi-cell interaction has also been lacking in the traditional in vitro models. The protocols to create human islet-like clusters have existed since 2004 (Segev et al. 2004). Unfortunately, the early protocols needed an in vivo xenotransplantation step to form functional glucose-responsive islet clusters (Rezania et al. 2012; Saber et al. 2018; Legøy et al. 2020b), which would make it challenging to perform chemical screen studies in this model. Now we have protocols able to generate SC-islets in vitro that closely mimic human islets metabolically (Balboa et al. 2022), thus making it an ideal model to assess how chemical exposure may alter the function of human islets.

Humans have a high chemical burden with a near-constant exposure to chemicals, with the main exposure routes being air pollution and digestion of processed food and drinks. This exposure rarely occurs in isolation. Instead, an individual is exposed to a complex mixture of chemicals, and many of these are EDCs. A cocktail of different EDCs could act on multiple endocrine axes and produce cumulative or amplified endocrine effects. It is therefore necessary to take chemical mixture into account when performing toxicological studies, as a mix might have non-linear, synergistic, or antagonistic effects and have a great adverse impact on human health (Kumar et al. 2020; Duh-Leong et al. 2023).

In the present study, we decided to create a low-dose mix of BPA, BPS, and *trans*-nonachlor. By using a low-dose exposure during differentiation of these compounds, we aimed to assess how a background exposure to these compounds can affect the maturation of SC-islets and their potential developmental impact. The main molecular mechanisms revealed by pathway analysis were connected to the cellular cycle and proliferation. We found an upregulation of key genes connected with proliferation, as well as an increased number of SC-islet cells co-expressing mKI67. Interestingly, very few studies reported these EDCs as proliferation-prone; rather, some reported an inhibition of proliferation. In cell lines of triple-negative breast cancers, exposure to a range of low doses of BPS, among them 1 nM, resulted in increased expression of stemness markers, like SOX2, OCT4, and Nanog, as well as migration and invasion of stem cells, but no increase in proliferation was observed (Yi et al. 2025). In human osteoblast, BPS exposure at 100 nM resulted in reduced proliferation and altered differentiation with lower mineralization (García-Recio et al. 2023). BPA has been reported to inhibit proliferation and cause cell cycle arrest in healthy prostate cells (Song et al. 2023) when using concentrations above 10 µM. Daily oral exposure of BPS in rats impairs neuronal differentiation and proliferation (Tiwari et al. 2024). Exposure to 10 µM of BPA has also been shown to alter differentiation of rat embryonic cardiomyoblasts (Escarda-Castro et al. 2021). The insecticide chlordane, of which trans-nonachlor is one compound, has been reported not to trigger proliferation in MCF-7 cells, even though it is considered a carcinogenic compound (Soto et al. 1995). Impairment of proliferation in the endocrine pancreas has also been observed following BPA exposure in combination with a high-fat diet in adult female mice (Oliveira et al. 2020). However, this picture changes for developmental exposure. Interestingly, when exposing pregnant female mice at Days 9 and 16 of gestation to BPA at a low dose of 10 μg/kg per day, an increased proliferation in pancreatic islets was observed in male offspring during the first month of age (García-Arévalo et al. 2016). To assess if the proliferation was induced by developmental impact, or as a direct effect of BPA, the authors performed an ex vivo treatment of male mice for 48 h, where they observed an increase in BrdU-positive beta cells (Boronat-Belda et al. 2020). The question remains if the BPA treatment during the developing pancreas directly causes an increase in proliferation, or if there is a delayed maturation with a retained pool of proliferative immature cells. Pancreatic beta cells proliferate during fetal and neonatal stages, an ability that is almost entirely absent in adult beta cells (Spears et al. 2021). There are some proposing that maybe proliferation inhibits differentiation (Scharfmann et al. 2014), or that the demand of the insulin production is what blocks the proliferation (Szabat et al. 2016). We show that SC-islets that had been treated with EDC during differentiation had a transcriptional landscape closer to immature cells and farther from human islets than the non-treated SC-islets. This observation supports the idea of a delayed maturation, but further studies are needed before a conclusion can be made.

**Fig. 6.**
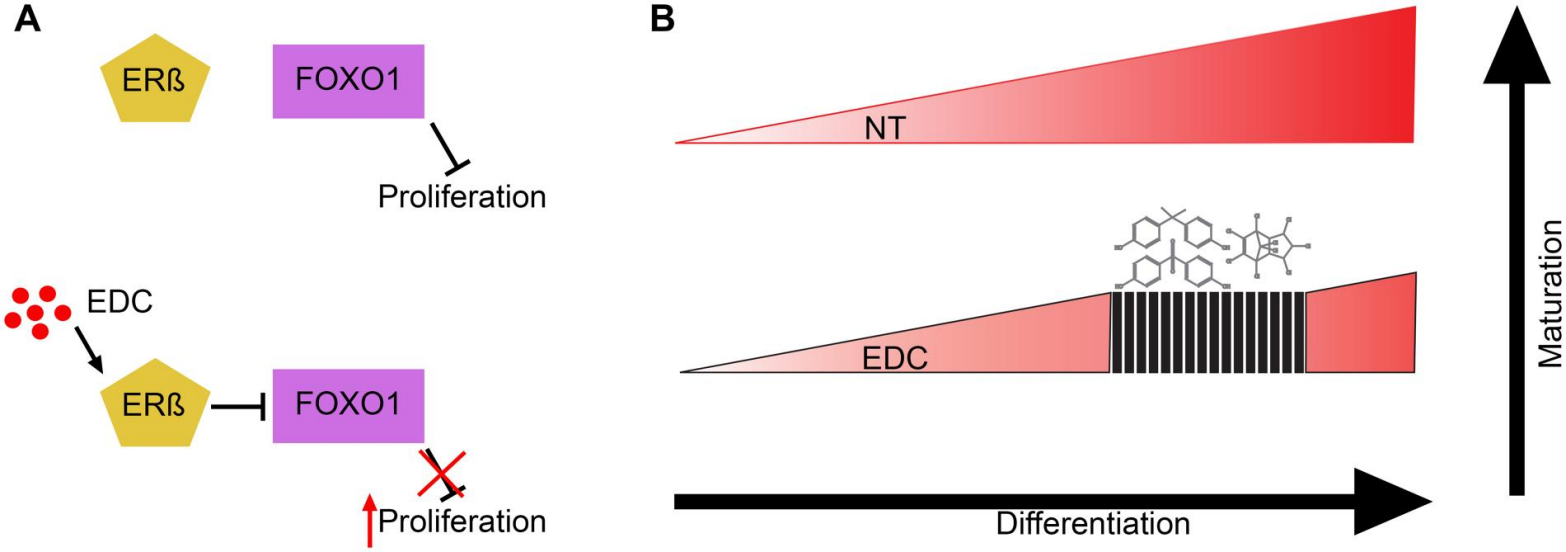
Suggested mechanism. A) Potential mechanism of EDC triggered inactivation of FOXO1 giving rise to increased proliferation signature. B) Illustration illustrating potential mechanism of action of EDC during differentiation.

In combination with the proliferative profile, we found a downregulation of FOXO1. As stated previously, FOXO1 is important in stress response, especially with regard to oxidative stress (Akasaki et al. 2014; Rodriguez-Colman et al. 2024). In addition, FOXO1 suppresses cell proliferation by regulating the transcription of key cell cycle modulators and contributes to the induction of cellular senescence (Adachi et al. 2007; Liu et al. 2008; Han et al. 2020). Further on, there are studies showing interactions between FOXO1 and the estrogen receptors (Kaya Okur et al. 2016; Yan et al. 2019), where activation of estrogen receptor-beta has been shown to inhibit FOXO1 and contribute to tumor growth (Ide et al. 2020). Given that increased proliferation induced by developmental exposure to BPA has been showed to be dependent on estrogen receptor-beta signaling (Boronat-Belda et al. 2020), one potential mechanism of increased proliferation signal could be induced through estrogen receptor and FOXO1 inhibition (Fig. 6A). However, further experiments are needed to confirm this mechanism.

EDC exposure during development can affect the hormone system homeostasis and transcriptional landscape, leading to adverse health outcomes (Singh et al. 2021). The differentiation of hiPSCs and hESCs toward SC-islets has great potential, as it guides the cells through different stages of development through stepwise addition of chemicals to mimic development (MacFarlane and Bruin 2021). However, this stepwise addition of chemicals will also push the cells through development in vitro, potentially masking the impact of EDC. EDC exposure could affect the development of multiple organs, which could affect the developmental signals received by cells in different cell pools during development, a signaling change that might be masked by a constant addition of developmental drivers during differentiation. On the other hand, this could also mean that the severity and adverse effects observed after EDC treatment might be more severe in an in vivo environment. More research is needed to assess the interplay of the differentiation and the EDC cocktails. Nevertheless, it has become more commonly accepted that developmental exposure to EDC chemicals can lead to small, subtle changes on molecular and cellular levels, leading to adverse health outcomes later in life, referred to as the Developmental Origins of Health and Disease hypothesis (Barker 2007; Heindel et al. 2015, 2017). Indeed, even with the differentiation protocol promoting SC-islet fate, the human-relevant level exposure of an EDC mix during a short critical window created less mature SC-islets with a blunted glucose response, even with subtle molecular changes.

In the last years, the idea of molecular alterations and dysfunction of pancreatic endocrine islets, and especially the insulin-producing beta cells, playing a key part in the initiation and progression of diabetes has been emerging. For example, in T2D, clinical data support that there might be an early beta-cell dysfunction that leads to hyperglycemia in individuals that develop T2D, and not the insulin resistance (Esser et al. 2020). A similar discussion also exists for T1D, with the overload hypothesis. Where exposure to environmental factors that cause beta-cell stress might lead to prolonged ER stress or trigger damage from the immune system (Rewers and Ludvigsson 2016; Howard 2018; Roep et al. 2021). Considering that cellular stress could lead to beta-cell dysfunction and diabetes, and exposure to EDC is known to cause cellular stress, SC-islets differentiation offers a promising model to investigate chemical exposure and its future implications for human health. Indeed, our SC-islets exposed to EDCs during differentiation, compared with non-treated, had molecular profiles with similarities to T2D human islets compared with non-diabetic human islets.

This present study has shown that hiPSCs-derived SC-islet differentiation can be a model to study the development impact of EDC on human pancreatic endocrine cell development and maturation. Treatment of a cocktail of BPA, BPS, and trans-nonachlor provided comparable results with in vivo studies of fetus exposure to BPA, further supporting the use of our model. Based on our findings, we would argue that exposure to EDC during differentiation delays the development and maturation during those stages, giving rise to less mature cells (Fig. 6B). Exposure studies at multiple stages during the differentiation protocol would be necessary to confirm these findings.

## Supplementary Material

kfaf163_Supplementary_Data

## Data Availability

The RNAseq data have been uploaded to the NCBI Gene Expression Omnibus (GEO) repository with the identifiers GSE285036, GSE291570, and GSE288932. The human islet data has been published previously (Legøy et al. 2020a) and is available through the identifier GSE141309. Proteomics data were uploaded to the Proteome exchange with the identifier PXD065436.
