# Supplementary material for: Endocrine-disrupting chemical exposure during differentiation alters the proliferation–maturation balance in stem-cell islets
Source: Toxicol Sci. 2025 Nov 20;209(1):kfaf163. doi: 10.1093/toxsci/kfaf163 (PMC12863212; doi:10.1093/toxsci/kfaf163)
Supplement: kfaf163_Supplementary_Data [file kfaf163_supplementary_data.docx]

**Endocrine Disrupting Chemical Exposure During Differentiation Alters the Proliferation-Maturation Balance in Stem-Cell islets**

June H. Gudmestad^1^, Lucas Unger^1^, Joao A. Paulo^2^, Luiza Ghila^1^, Thomas A. Legøy^1*^

**Supplementary Material**


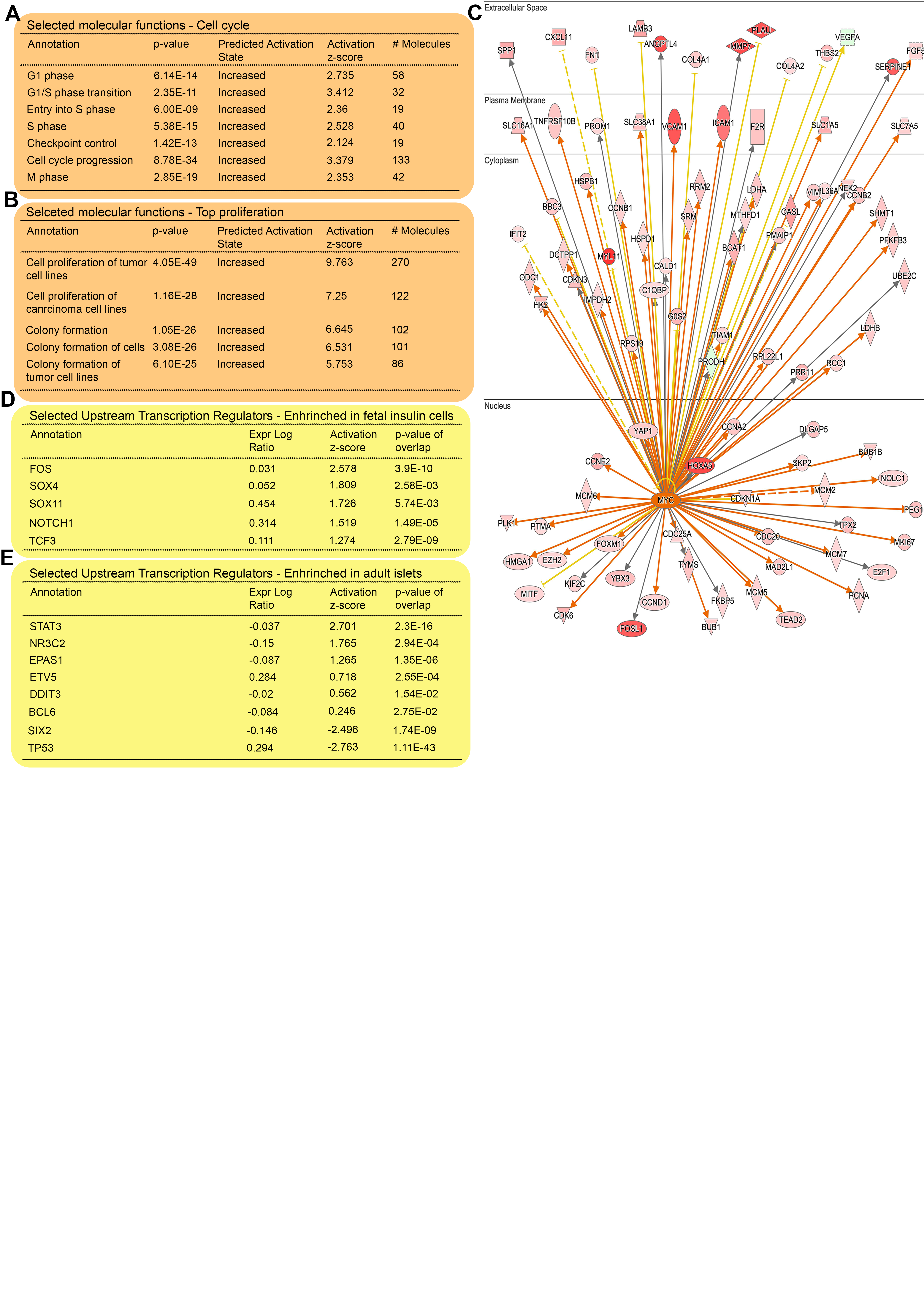


Supplementary Figure 1

A,B) Table showing the top selected molecular functions related to A) cell cycle or B) proliferation, based on pathway analysis of DEGs between EDC and NT. C) Regulatory network centered around MYC based on pathway analysis of DEGs between EDC and NT. D) Table showing the top upstream transcription regulators that has been shown to be upregulated in fetal insulin cells. E) Table showing the top upstream transcription regulators that has been shown to be enriched in adult islets. All based on pathway analysis of DEGs between EDC and NT.


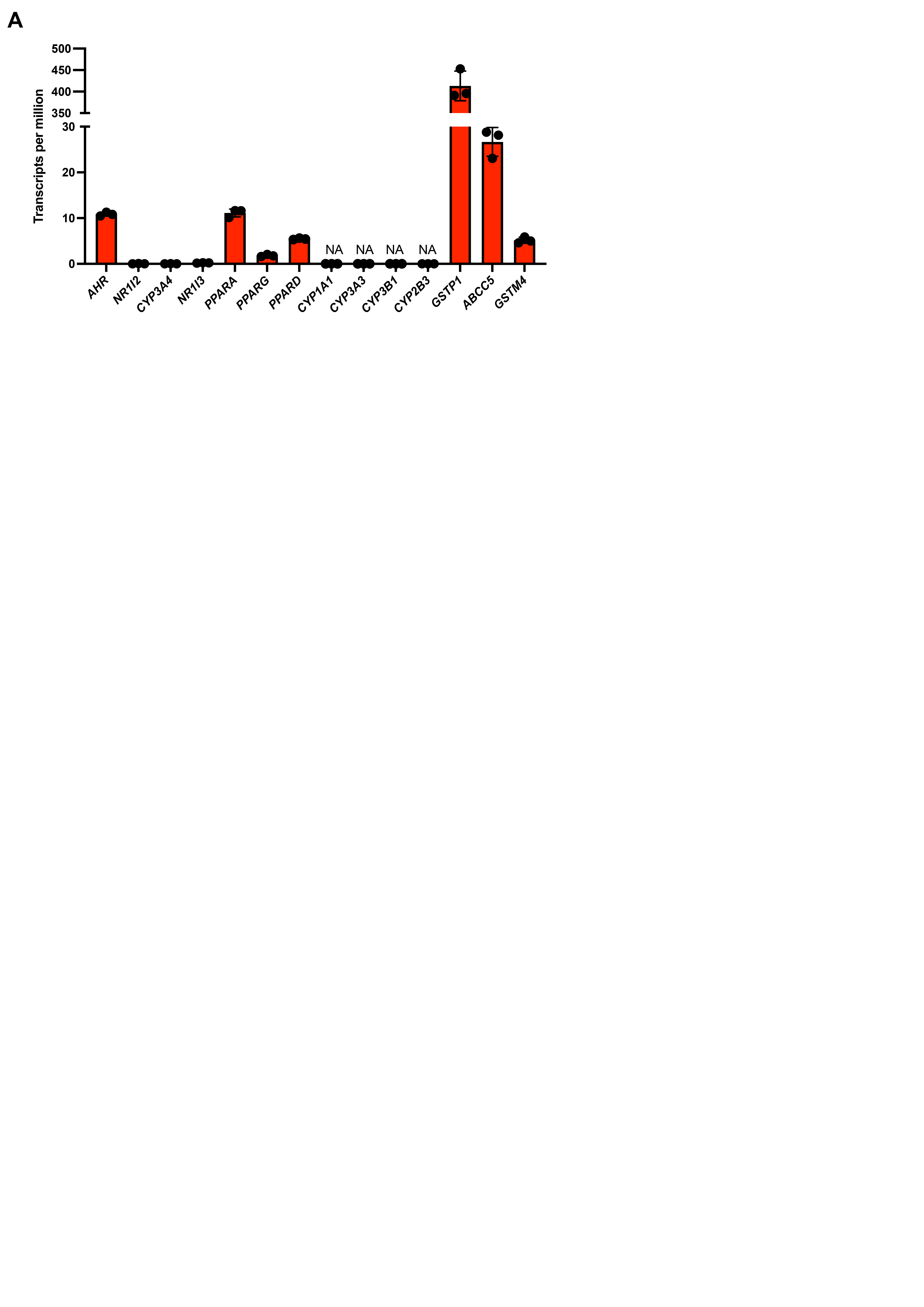


Supplementary Figure 2

A) Average transcripts per million (RNAseq) from EDC group of detoxification genes, graph data are shown as average ± SD.

**Supplement table 1**

| **Differentiation media** | | | | |
| --- | --- | --- | --- | --- |
| **Name** | **Reagent** | **Final Concentration** | **Provider** | **Product nr.** |
| Diff 1 | MCDB131 | | Fisher Scientific | 11513407 |
|  | Penicillin-Streptomycin | 0.1 % | Sigma Aldrich | P4333 |
|  | NaHCO_3_ | 1.5 g/L | Fisher Scientific | 12675057 |
|  | Glutamax | 1x | Fisher Scientific | 13462629 |
|  | Glucose | 10 mM | Sigma Aldrich | G7021 |
|  | BSA | 0.5 % | Sigma Aldrich | A7030 |
| Diff 2 | MCDB131 |  | Fisher Scientific | 11513407 |
|  | Penicillin-Streptomycin | 0.1 % | Sigma Aldrich | P4333 |
|  | NaHCO_3_ | 2.5 g/L | Fisher Scientific | 12675057 |
|  | Glutamax | 1x | Fisher Scientific | 13462629 |
|  | Glucose | 10 mM | Sigma Aldrich | G7021 |
|  | BSA | 2 % | Sigma Aldrich | A7030 |
|  | ITS-X | 0.5x | Fisher Scientific | 10524233 |
| Diff 3 | MCDB131 | | Fisher Scientific | 11513407 |
|  | Penicillin-Streptomycin | 0.1 % | Sigma Aldrich | P4333 |
|  | NaHCO_3_ | 1.5 g/L | Fisher Scientific | 12675057 |
|  | Glutamax | 1x | Fisher Scientific | 13462629 |
|  | Glucose | 20 mM | Sigma Aldrich | G7021 |
|  | BSA | 2 % | Sigma Aldrich | A7030 |
|  | ITS-X | 0.5x | Fisher Scientific | 10524233 |
|  | ZnSO_4_ | 10 uM | Sigma Aldrich | Z0251 |
|  | Heparin | 10 ug/ml | Sigma Aldrich | H3149 |
| Diff 4 | CMRL 1066 | | VWR | 15-110-CV |
|  | Penicillin-Streptomycin | 0.1 % | Sigma Aldrich | P4333 |
|  | Sodium pyruvate | 0.5 mM | Fisher Scientific | 12675057 |
|  | Glutamax | 1x | Fisher Scientific | 13462629 |
|  | BSA | 2 % | Sigma Aldrich | A7030 |
|  | ITS-X | 0.5x | Fisher Scientific | 10524233 |
|  | ZnSO_4_ | 10 uM | Sigma Aldrich | Z0251 |
|  | Heparin | 10 ug/ml | Sigma Aldrich | H3149 |
|  | Lipid concentrate | 1x | Fisher Scientific | 11548846 |
|  | Trace element A | 1x | Fisher Scientific | 15333641 |
|  | Trace element B | 1x | Fisher Scientific | 15343641 |

**Supplement table 2**

| **Supplements** | | | | |
| --- | --- | --- | --- | --- |
| **Name / concentration** | **Chemical** | **Concentration** | **Provider** | **Product nr.** |
| Supplement 3  2500x | SANT1 | 0.625 mM | Sigma Aldrich | S4572 |
|  | Retinoic acid | 2.5 mM | Sigma Aldrich | R2625 |
|  | LDN-193189 | 0.25 mM | Tebu-Bio | 24804-0074 |
|  | PKC Activator V | 0.5 mM | Sigma Aldrich | 565740 |
|  | In DMSO | | | |
| Supplement 4  2500x | SANT1 | 0.625 mM | Sigma Aldrich | S4572 |
|  | Retinoic acid | 0.25 mM | Sigma Aldrich | R2625 |
|  | LDN-193189 | 0.5 mM | Tebu-Bio | 24804-0074 |
|  | PKC Activator V | 0.25 mM | Sigma Aldrich | 565740 |
|  | In DMSO |  |  |  |
| Supplement 5  1500x | SANT1 | 0.625 mM | Sigma Aldrich | S4572 |
|  | Retinoic acid | 0.25 mM | Sigma Aldrich | R2625 |
|  | LDN-193189 | 0.5 mM | Tebu-Bio | 24804-0074 |
|  | GC1 | 1.5 mM | Sigma Aldrich | SML1900 |
|  | GSIXX | 0.15 mM | Sigma Aldrich | 565789 |
|  | Alk5 inhibitor II | 15 mM | Enzo Life Science | ALX-270-445 |
|  | In DMSO | | | |
| Supplement 6  200x | LDN-193189 | 0.2 mM | Tebu-Bio | 24804-0074 |
|  | GC1 | 2 mM | Sigma Aldrich | SML1900 |
|  | GSIXX | 0.2 mM | Sigma Aldrich | 565789 |
|  | Alk5 inhibitor II | 15 mM | Enzo Life Science | ALX-270-445 |
|  | In DMSO | | | |

**Supplement table 3**

| **Differentiation cocktail** | | | | | |
| --- | --- | --- | --- | --- | --- |
| Day(s) | Medium | Diff Chemical | Final Concentration | Provider | Product nr. |
| 1 | Diff 1 | Activin A | 100 ng/ml | Peprotech | 120-14 |
|  |  | GSK inhibitor | 3 uM | Selleck chemicals | S2924 |
| 2 | Diff 1 | Activin A | 100 ng/ml | Peprotech | 120-14 |
|  |  | GSK inhibitor | 0.3 uM | Selleck chemicals | S2924 |
| 3 | Diff 1 | Activin A | 100 ng/ml | Peprotech | 120-14 |
| 4-6 | Diff 1 | FGF7 | 50 ng/ml | Peprotech | 100-19 |
|  |  | Ascorbic acid | 0.25 mM | Sigma Aldrich | A4544 |
| 7-8 | Diff 2 | Supplement 3 | 1x |  |  |
|  |  | FGF7 | 50 ng/ml | Peprotech | 100-19 |
|  |  | Ascorbic acid | 0.25 mM | Sigma Aldrich | A4544 |
| 9-12 | Diff 2 | Supplement 4 | 1x |  |  |
|  |  | FGF7 | 2 ng/ml | Peprotech | 100-19 |
|  |  | Ascorbic acid | 0.25 mM | Sigma Aldrich | A4544 |
|  |  | Activin A | 10 ng/ml | Peprotech | 120-14 |
|  |  | hEGF | 100 ng/ml |  |  |
|  |  | Y-27632 dihydrochloride | 10 uM | Tebubio | 10-2301 |
|  |  | Nicotinamide | 10 mM |  |  |
| 13-16 | Diff 3 | Supplement 5 | 1x |  |  |
|  |  | Human Betacellulin Recombinant Protein | 20 ng/ml | PeproTech | 100-50 |
|  |  | Y-27632 dihydrochloride | 10 uM | Tebubio | 10-2301 |
| 17-23 | Diff 3 | Supplement 6 | 1x |  |  |
| 24-38 | Diff 4 | T3 | 10 mM | Sigma Aldrich | T6397 |
|  |  | N-acetyl cysteine | 1 mM | Merck | A9165 |
|  |  | ZM-447439 | 0.5 uM | Selleck chemicals | S1103 |
